# Nontuberculous Mycobacterial Infection after Fractionated CO_2_ Laser Resurfacing

**DOI:** 10.3201/eid1903.120880

**Published:** 2013-03

**Authors:** Donna A. Culton, Anne M. Lachiewicz, Becky A. Miller, Melissa B. Miller, Courteney MacKuen, Pamela Groben, Becky White, Gary M. Cox, Jason E. Stout

**Affiliations:** Author affiliations: University of North Carolina, Chapel Hill, North Carolina, USA (D.A. Culton, A.M. Lachiewicz, M.B. Miller, C. MacKuen, P. Groben, B. White);; Duke University Medical Center, Durham, North Carolina, USA (B.A. Miller, G.M. Cox, J.E. Stout)

**Keywords:** tuberculosis and other mycobacteria, nontuberculous mycobacterial infection, nontuberculous mycobacteria, NTM, Mycobacterium chelonae, Mycobacterium abscessus, bacteria, fractionated CO_2_ laser resurfacing, cosmetic techniques

## Abstract

Nontuberculous mycobacteria are increasingly associated with cutaneous infections after cosmetic procedures. Fractionated CO_2_ resurfacing, a widely used technique for photorejuvenation, has been associated with a more favorable side effect profile than alternative procedures. We describe 2 cases of nontuberculous mycobacterial infection after treatment with a fractionated CO_2_ laser at a private clinic. Densely distributed erythematous papules and pustules developed within the treated area within 2 weeks of the laser procedure. Diagnosis was confirmed by histologic analysis and culture. Both infections responded to a 4-month course of a multidrug regimen. An environmental investigation of the clinic was performed, but no source of infection was found. The case isolates differed from each other and from isolates obtained from the clinic, suggesting that the infection was acquired by postprocedure exposure. Papules and pustules after fractionated CO_2_ resurfacing should raise the suspicion of nontuberculous mycobacterial infection.

Nontuberculous mycobacteria (NTM) are increasingly associated with cutaneous and soft tissue infections after cosmetic and spa procedures, such as liposuction, mammoplasty, blepharoplasty, mesotherapy, and whirlpool footbaths during pedicures (*1*–*5*). These infections are often difficult to diagnose, resulting in major treatment delays (*4*,*6*). Fractionated CO_2_ laser resurfacing is a widely used cosmetic procedure that minimizes the appearance of rhytides (skin wrinkles) and acne scars, and compared with older laser procedures, fractionated CO_2_ resurfacing is associated with less downtime and a lower rate of infectious and noninfectious complications (*7*–*9*). Although fractionated CO_2_ laser therapy is associated with decreased rates of postprocedure infection, infections such as herpes simplex virus, bacterial, and candidal infections have been reported (*8*–*10*). Palm et al. recently reported the first case of NTM infection caused by *Mycobacterium chelonae* after treatment with a fractionated CO_2_ laser for facial resurfacing (*11*). Given the length of time from the procedure to the diagnosis (≈2 months), a source of NTM infection was not sought.

We report 2 additional cases of NTM infection after treatment with fractionated CO_2_ resurfacing at the same private clinic and an extensive environmental investigation to identify a source of infection. This study received formal exemptions from review by the Institutional Review Boards of the University of North Carolina and Duke University Medical Center.

## Case-Patient 1

**Figure 1:**
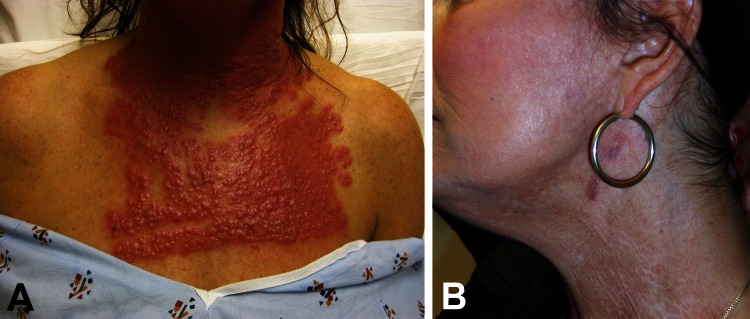
A) Neck and chest of a 53-year-old woman (case-patient 1) 14 days after fractionated CO_2_ laser resurfacing. B) Neck of the patient after 5 months of multidrug therapy and pulsed dye laser treatment.

A 53-year-old woman had multiple erythematous papules and pustules densely distributed over her face, neck, and chest (Figure 1, panel A) 2 weeks after receiving fractionated CO_2_ laser resurfacing (Solta Medical Inc., Hayward, CA, USA). Before laser resurfacing, the patient began a prophylactic 7-day course of valacyclovir because of a history of recurrent herpes labialis. Immediately before the procedure, the patient’s skin was cleansed with 70% isopropanol. Topical lidocaine/tetracaine ointment was applied to the skin for topical anesthesia, followed by intraoral nerve block and tumescent anesthesia for the face only. The neck and chest were treated at 40 mJ (treatment level 7 mJ) and 25% coverage, the forehead at 60 mJ (treatment level 9 mJ) and 35% coverage, and the nose and cheeks at 70 mJ (treatment level 9 mJ) and 35% coverage (total 10.46 kJ). Immediately after the procedure, the patient’s skin was cleansed with sterile saline, and emollient was applied. Postprocedure home wound care consisted of vinegar solution (vinegar diluted with bottled water) applications once a day and avoidance of showering, scrubbing, and cosmetics for 72 h.

Ten days post–laser treatment, erythematous papules and pustules developed over the face, neck, and chest of the patient. Outpatient treatment was initiated with oral fluconazole, doxycycline, and valacyclovir for presumed fungal, staphylococcal, or disseminated herpes simplex virus infection. Because of extensive pruritus, the patient was given locoid lipocin (0.1% hydrocortisone butyrate) and a tapered dose of prednisone for possible allergic contact dermatitis. She reported adherence to instructions to avoid showering and washing her face with tap water for 72 h after the procedure. However, she was exposed to dust from sanding she did at home during the week after the procedure. She did not show improvement over the next 2 days and, after a low-grade fever developed, was hospitalized and received intravenous acyclovir therapy for presumed disseminated herpes simplex virus infection.

**Figure 2:**
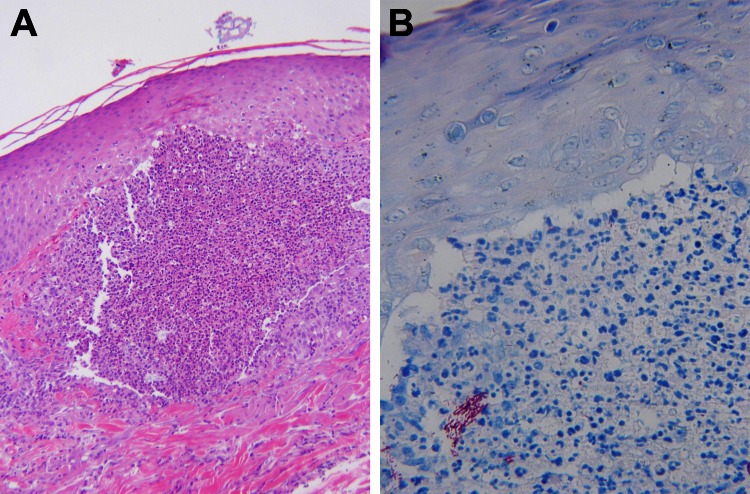
Skin biopsy specimens of a 53-year-old woman (case-patient 1) after fractionated CO_2_ laser resurfacing. A) Hematoxylin and eosin–stained and B) Ziehl-Neelsen acid-fast–stained sections show a tiny superficial microabscess surrounded by sparse granulomatous inflammation. Several groups of acid-fast organisms can be seen at the lower left of panel B. Original magnifications: 400× in A and 600× in B.

When the patient was hospitalized, lesions were nearly confluent over her neck and chest and scattered over her face but limited to areas treated with the CO_2_ laser. PCR results for herpes simplex virus, varicella zoster virus, and fungal cultures were negative. Gram staining showed polymorphonuclear leukocytes and gram-variable rods. Two skin biopsy specimens demonstrated multiple, tiny foci of suppurative granulomatous dermatitis with elastophagocytosis (Figure 2, panel A) and numerous long acid-fast rods that were gram positive (Figure 2, panel B).

The patient was given empiric treatment for nontuberculous mycobacterial infection with intravenous tigecycline combined with oral moxifloxacin and azithromycin. Two weeks later, tissue culture of her lesions grew *M. abscessus*. Drug susceptibility testing showed resistance to moxifloxacin, amoxicillin/clavulanate, tobramycin, trimethoprim/sulfamethoxazole, ciprofloxacin, doripenem, linezolid, and doxycycline, and susceptibility to azithromycin, amikacin, kanamycin, imipenem, cefoxitin, and tigecycline.

One month after initiation of the multidrug regimen, repeat culture of a persistent pustule on her face again grew *M. abscessus*. Treatment with tigecycline and moxifloxacin was stopped after 2 months and treatment with azithromycin was stopped after 5 months because the patient showed clinical improvement. Scarring and dyspigmentation were observed. Thus, for cosmesis, she subsequently underwent a series of procedures with a pulsed dye laser (Figure 1, panel B).

## Case-Patient 2

**Figure 3:**
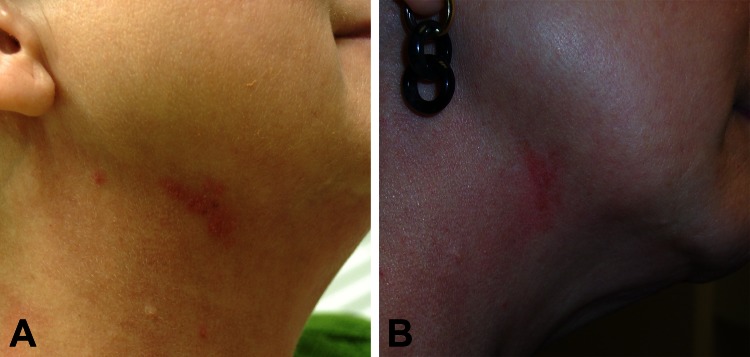
A) Right lateral neck of a 52-year-old woman (case-patient 2) 9 days after fractionated CO_2_ laser resurfacing. B) Neck of the patient after 4 months of multidrug therapy.

A 52-year-old woman underwent fractionated CO_2_ laser resurfacing of the neck at the same private clinic as case-patient 1 (66 days after case-patient 1 was treated). After case-patient 1 was treated, major changes were made in the treatment protocol to make the procedure sterile. Treatment was performed at 30 mJ (treatment level 7) with 25% coverage (total 2.96 kJ) but otherwise as for case-patient 1. Nine days after the procedure, painful pustular lesions developed within the treated area but primarily on the right neck (Figure 3, panel A). The patient reported adherence with instructions to avoid washing with tap water for 72 hours after the procedure and denied any other exposures. Treatment was initiated with valacyclovir, cephalexin, and topical antimicrobial drugs.

After this patient did not show improvement, a biopsy specimen from a lesion showed suppurative and granulomatous dermatitis, which suggested NTM infection. Empiric treatment for NTM infection was initiated with azithromycin and moxifloxacin; some improvement in the lesions was subsequently observed. The organism was identified as *M. chelonae*. Drug susceptibility testing showed resistance to cefoxitin and trimethoprim/sulfamethoxazole; intermediate susceptibility to ciprofloxacin; and susceptibility to amikacin, clarithromycin, linezolid, and tobramycin. Treatment was continued with azithromycin and moxifloxacin for 4 months and clinical improvement was observed (Figure 3, panel B).

## Epidemiologic Investigation

After case 1 was detected, an epidemiologic investigation was requested by the physician (dermatologist) who had performed the laser resurfacing to investigate possible sources of the infection within the clinic. The investigation was initiated 27 days after the procedure was performed. We interviewed the dermatologist and reviewed all steps of the procedure. Several items were obtained and cultured for nontuberculous mycobacteria: a multiuse jar of lidocaine/tetracaine ointment, a multiuse jar of emollient, a multiuse vial of 1% lidocaine used for nerve blockimg, a nonsterile package of gauze used to apply ointments to patients’ skin, and a multiuse vial of sodium bicarbonate. Small amounts of samples were swabbed onto 7H10 Middlebrook medium supplemented with an additional 5 µg/mL of malachite green by using a sterile swab. A gauze square was immersed in ≈250 mL sterile water and agitated vigorously for ≈1 min. The water was then filtered through a 0.4-μ sterile filter, and the filter was plated directly onto the 7H10 Middlebrook medium. Approximately 0.1 mL of each of the other samples was placed directly onto a 7H10 Middlebrook medium plate and spread over the plate by using a sterile loop. Plates were incubated at 30°C, and no growth was observed on any plates after 3 months of incubation.

Although no obvious source of infection was identified, several changes to the routine fractionated CO_2_ resurfacing procedure were made after case 1 was detected. These changes included use of sterile gloves; sterile gauze; sterile tongue depressors for application of topical lidocaine/tetracaine ointment; conversion to single-use vials of lidocaine, epinephrine, and sterile saline used for tumescent anesthesia; and single-use postprocedure emollient. Chloroxylenol (3%) was also added in addition to 70% isopropanol for preprocedure cleansing. Postprocedure wound care was not changed.

After case 2 was detected, a second site visit was arranged 46 days after the procedure was performed. The dermatologist was interviewed again, and a complete sham procedure was performed while the investigators observed. Samples were collected from the 3% chloroxylenol, hand scrub, multiuse sodium bicarbonate vial, and single-use lidocaine/tetracaine ointment vial. The suction canister, connection tubing, and smoke filter were removed from the machine and cultured. Copious amounts of skin debris were identified in the long and short tubes of the connection tubing, along with an ≈2 to 3–cm layer of skin debris on top of the smoke filter. Environmental swabs of the countertops and walls in the procedure room were also collected. Water specimens (≈250 mL) were collected from the taps in the staff and patient bathrooms in the clinic (there was no water source in the procedure room or any other nearby procedure rooms) and from a fountain in the hallway. Patients were routinely instructed to wash the area with soap and water before coming to the office (i.e., they did not wash in the patient sink in the office). 

The ointments were plated directly onto Lowenstein-Jensen (LJ) and modified 7H10 medium. The environmental swab was plated directly onto LJ medium only. Two 1 × 3–cm pieces of the paper filter from the filter canister were plated directly onto LJ medium. In addition, the multiuse lidocaine/tetracaine ointment from the first site visit was plated directly onto LJ medium by using a sterile swab. Approximately 100 mL of sterile water were passed through the canister/short tube and the long tube from the apparatus and collected in sterile bottles. The resultant suspensions were brown and contained large quantities of skin debris. Approximately 40 mL of each water sample and 10 mL of the sodium bicarbonate were passed through 0.4-μ filters; each specimen was processed in duplicate. One filter from each specimen was then plated directly onto LJ medium, and the other filter was plated onto malachite green–supplemented 7H10 medium. Medium plates were incubated at 30°C. After 1 week, mycobacterial colonies were identified on the medium containing the filtrate from the connector tubing and several of the tap water specimens (staff bathroom and patient bathroom).

Species identification of all isolates was performed at the Microbiology Laboratory of the University of North Carolina by using 16S rRNA and heat shock protein 65 gene sequencing. Results of sequence analysis showed that the 2 patient isolates did not match. They were identical by sequencing of part of the 16S rRNA gene but differed by heat shock protein 65 sequencing; the organism isolated from case-patient 1 was *M. abscessus* and that from case-patient 2 was *M. chelonae*. Analysis of clinic water isolates showed several different mycobacterial organisms. Four colony morphologies were isolated from the tap water in the patient bathroom, 2 of which were identified as *M. mucogenicum*, 1 as *M. obuense/aurum*, and 1 as *M. chelonae*. Three colonies isolated from the tap in the staff bathroom were identified as *M. mucogenicum*. The isolate from the large tubing leading to the smoke filter was *M. smegmatis*, which was not a match with either patient isolate.

Pulsed-field gel electrophoresis was performed at the University of Texas at Tyler to compare the *M. chelonae* isolate from the case-patient 2 with the isolate from the patient bathroom in the dermatology clinic. The 2 isolates did not match.

Environmental investigation of the homes of the 2 patients was not conducted because case-patient 1 refused and sampling of the home of case-patient 2 was not attempted. The bottles of vinegar used for postprocedure cleansing were not available for either patient. Neither patient had undergone fractionated CO_2_ laser resurfacing before the procedures described.

## Conclusions

NTM are environmental organisms that are increasingly associated with systemic and cutaneous disease in humans. NTM-induced cutaneous disease typically occurs after injections, such as tattoos, botulinum toxin, and mesotherapy, or after minor surgical procedures in which breaks in the skin barrier occur (*1*,*3*,*5*,*12*–*15*). These organisms also have been associated with whirlpool footbaths before pedicures (*2*,*4*,*16*). In these cases, shaving before a whirlpool footbath was associated with increased rates of infection, presumably caused by microbreaks in the skin, which enable easy inoculation (*2*,*16*,*17*). NTM are ubiquitous in soil and water and have been detected in municipal water sources throughout the United States (*16*,*18*,*19*). They are also found in biofilms and, in the whirlpool footbath associated cases, seem to thrive in nutrient-rich water contaminated by skin debris, which accumulates on bath filters (*2*,*4*,*16*,*17*).

Eradication can be difficult because these organisms are resistant to most disinfectants (*4*,*20*,*21*). Cutaneous infection with NTM is most often caused by *M. marinum* and rapidly growing mycobacteria that belong to 1 of 3 species groups: *M. abscessus*, *M. chelonae*, and *M. fortuitum* (*22*–*24*). Diagnosis is difficult and often requires histologic evaluation and tissue samples for culture. Delays in diagnosis are common and can lead to delays in treatment (*6*). Species identification can be difficult and requires sequencing of multiple genes because of homology between *Mycobacterium* spp. family members (*25*–*27*). More than 120 NTM species have been identified, including ≈30 isolates in the past decade whose names might be unfamiliar to many clinicians (*28*). These organisms are also resistant to many antimicrobial drugs, a factor that complicates treatment.

In the past 5 years, fractionated CO_2_ laser resurfacing has become the preferred procedure for rhytides, photodamage, and acne scars (*9*). This procedure combines the efficacy of ablative laser resurfacing with a more favorable side effect profile than traditional ablative therapy. Studies have shown a high degree of safety and efficacy and lower rates of hypopigmentation, scarring, and infectious complications (*7*–*10*,*29*). This technology is based on the principle of creating narrow columns of tissue damage known as microthermal treatment zones, which are evenly distributed over the treated area. Localized epidermal necrosis and collagen denaturation occur in each column but the stratum corneum remains intact. Decreased disruption of the epidermal barrier and areas of viable tissue around each microthermal treatment zone enable more rapid healing and reduce the risk of infection.

Although infections with fractionated CO_2_ laser therapy are less common than with traditional ablative laser, they do occur (*10*). Infection with herpes simplex virus was reported in 1.7% of all cases and in 4.6% of cases in which the patient had a history of oral herpes but no antivirus prophylaxis was given (*8*). Bacterial complications, including *Staphylococcus* spp. and *Pseudomonas* spp. infections, and *Candida* spp. infections have also been reported, although at low rates (*10*).

Palm et al. reported the first case of NTM infection after fractionated laser resurfacing (*11*). The causative agent was identified as *M. chelonae* 3 months after the onset of acneiform eruptions. The patient received multidrug treatment and showed some clinical improvement. She eventually underwent therapy with a pulsed dye laser and showed a decrease in erythema and scarring. A possible source of NTM infection was not sought in this case.

We report 2 additional cases of NTM infection with *M. abscessus* and *M. chelonae* after fractionated CO_2_ laser resurfacing. Both patients showed development of erythematous papules and pustules ≈10–14 days after the procedure, but the extent of skin involvement varied between the 2 patients. For both patients, a diagnosis was made within 1–2 weeks by histologic examination and tissue culture. Early treatment with multidrug therapy specific for the most likely mycobacterial pathogens was initiated while susceptibility testing was performed. In both patients, treatment was continued for >4 months.

Results of a thorough epidemiologic investigation showed no evidence that transmission of the NTM infections occurred during the fractionated resurfacing procedure. The 2 patient isolates belonged to 2 different species, and neither matched the isolate obtained from the fractionated laser apparatus. Furthermore, none of the isolates from environmental water at the clinic matched either patient isolate. Although the absence of evidence does not definitively rule out common source transmission during the procedure, it does make it more likely that infection occurred elsewhere after the procedure.

The source of these infections remains unclear. It is possible that the causative NTM isolates were transiently present in the environment but were not detected because of lag times between procedures and environmental investigations. Furthermore, limitations of environmental sampling and culture for mycobacteria did not enable us to rule out a common source of infection at the time of the procedure. Detailed environmental sampling of the home was not permitted by the first patient and was not sought for the second patient. Several alternative environmental sources for infection are possible (aerosols from sinks, toilets, water fountains, and sanding dust for case-patient 1). Although there was no evidence to support exposure during the fractionated laser procedure, an NTM species was isolated from the tubing of the machine. Therefore, the tubing leading to the smoke filter is a potential reservoir for NTM because it is changed infrequently and can contain skin debris within the corrugated tubing.

Patients should be explicitly advised of the risk for NTM infection after fractionated laser resurfacing, and physicians should be highly suspicious of such infections during the postprocedure period. Although incubation periods reported for postprocedure NTM have been reported as 9–10 days, other cutaneous NTM infections may be found <3 months after the presumed exposure (*16*). Thus, late manifestations might be possible. Biopsy specimens for histologic evaluation and tissue culture are critical for making an accurate diagnosis. Suppurative neutrophilic and granulomatous dermatitis should raise suspicion for NTM infection, even if results of staining for acid-fast bacilli are negative. As shown by these cases and the case described by Palm et al. (*11*), identification of gram-positive rods during routine histologic examination might suggest NTM infection because these organisms can be weakly gram positive.

Empiric therapy specific for NTM should be considered while awaiting biopsy and culture results for patients with suspicious lesions. However, prophylactic therapy before or after the procedure with active agents against NTM is not recommended. The efficacy of such treatment in preventing infection remains unknown, and the risk for antimicrobial drug–associated side effects likely outweighs any theoretical benefit. Although there is no standard treatment for cutaneous NTM infections, multidrug therapy is usually necessary to minimize the development of drug resistance. Antimicrobial drug susceptibility testing should be conducted to tailor therapy, and treatment should be continued for 4–6 months.

When these infections occur, systematic observation of the procedure should be performed. Specifically, attention should be paid to any liquids or ointments that may contact the skin of a patient during or just after the procedure (particularly multiuse vials or containers) and the proximity of the procedure room to potentially aerosol-generating water sources. Environmental sampling with mycobacterial culture of such liquids seems to be a reasonable first step in identifying a source (although it did not identify a source in this study).

Furthermore, strict postprocedure wound care is critical to minimize risk for NTM infection. It is prudent to advise patients to avoid any municipal water sources for the first 72 h after the procedure (although this time interval is arbitrary). Bottled water, which may not be sterile, could harbor small amounts of NTM. Use of sterile water or sterile saline for postprocedure cleansing is recommended. First and foremost, physicians must remain aware of this potential complication of fractionated laser resurfacing and be highly suspicious even if initial histologic and culture results do not identify microbial pathogens.
